# Supplementary material for: Inactivation of the type I interferon pathway reveals long double‐stranded RNA‐mediated RNA interference in mammalian cells
Source: EMBO J. 2016 Nov 4;35(23):2505–18. doi: 10.15252/embj.201695086 (PMC5167344; doi:10.15252/embj.201695086)
Supplement: Supplementary file 1 — Appendix [file EMBJ-35-2505-s001.pdf]

Appendix

Inactivation of the type I interferon pathway reveals long double stranded RNA-mediated RNA interference in mammalian cells

Pierre V. Maillard[1], Annemarthe G. Van der Veen[1], Safia Deddouche-Grass[1†], Neil C. Rogers[1], Andres Merits[2] & Caetano Reis e Sousa[1]

[1] Immunobiology Laboratory, The Francis Crick Institute, 1 Midland Road, London NW1 1AT, UK.

[2] Institute of Technology, University of Tartu, 50411 Tartu, Estonia.

[†]Present address: Open Innovation Access Platform, Sanofi Strasbourg, 16 rue d'Ankara, 67080 Strasbourg France.

Appendix table of contents

Appendix Figures S1-S7
Appendix Figure legends S1-S7
Appendix Table S1

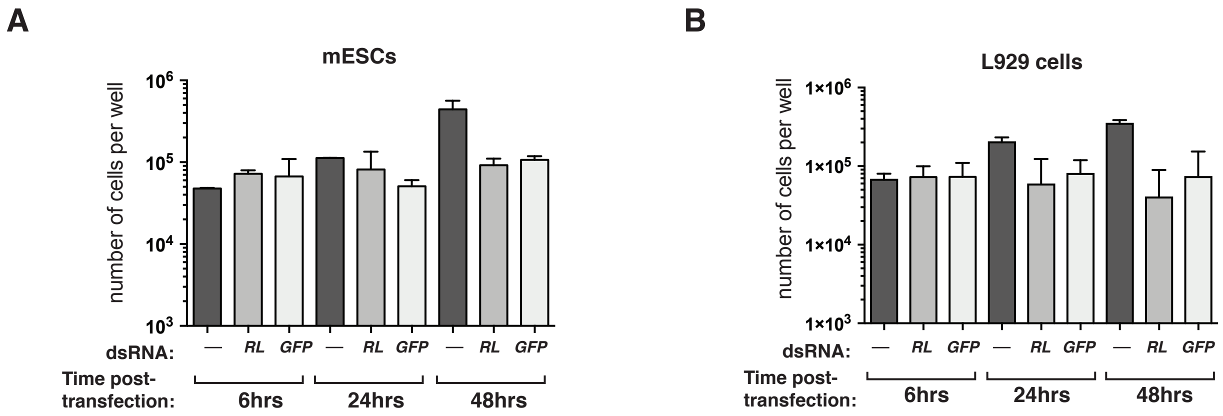

Appendix Figure S1

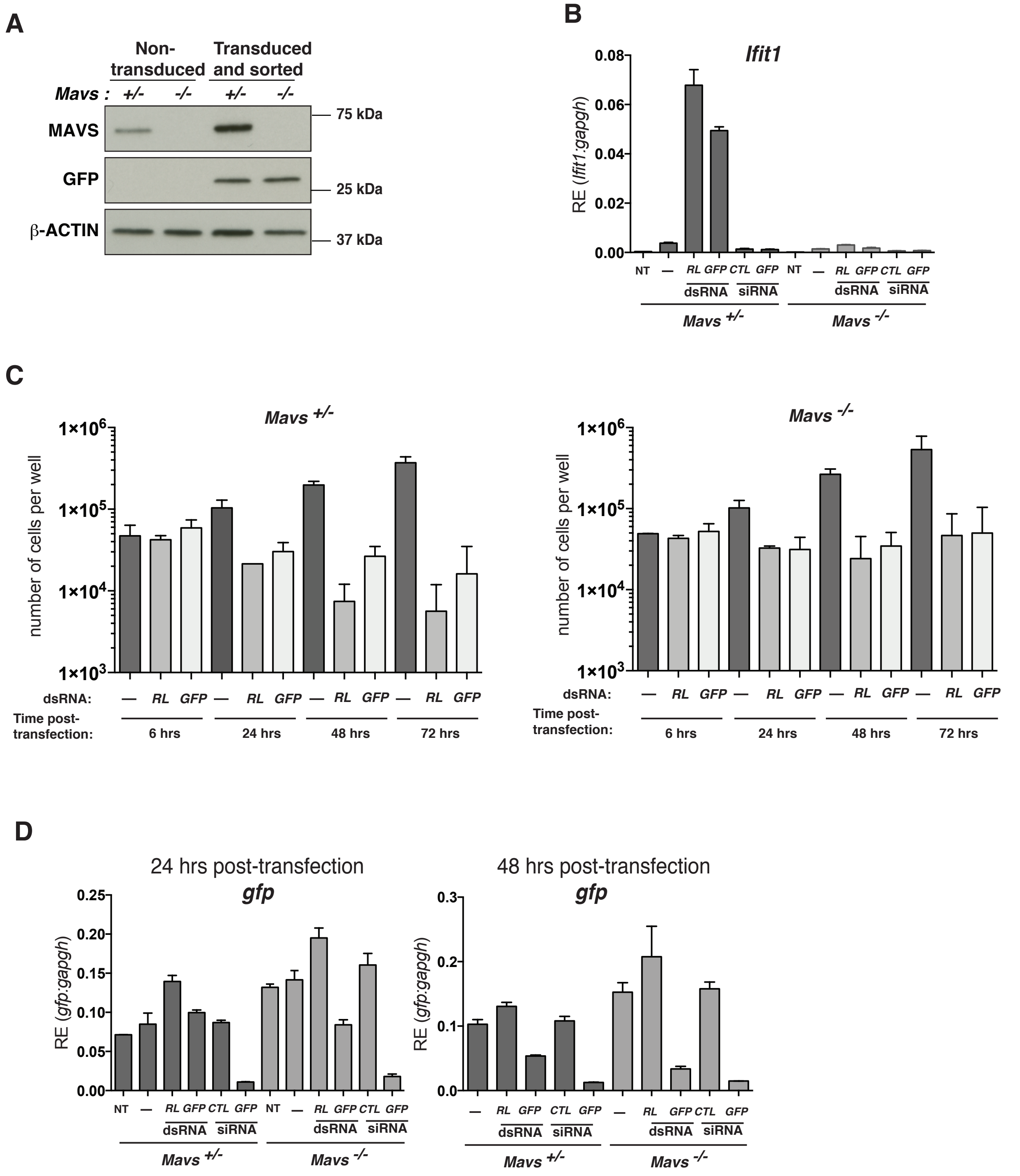

Appendix Figure S2

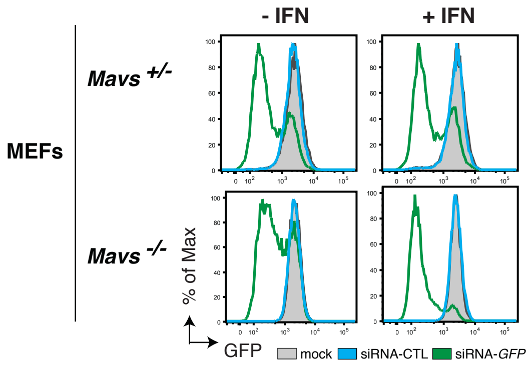

Appendix Figure S3

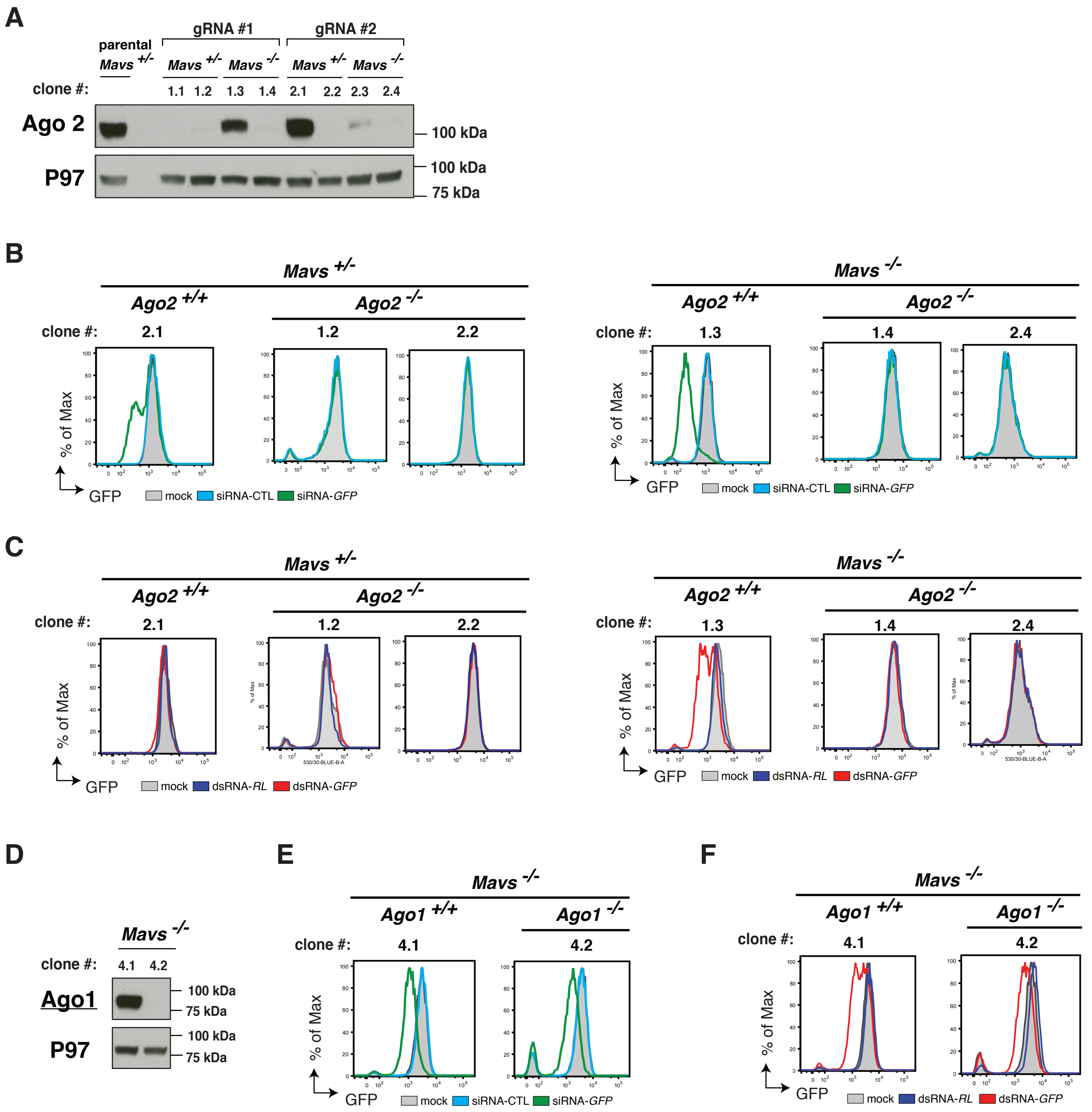

Appendix Figure S4

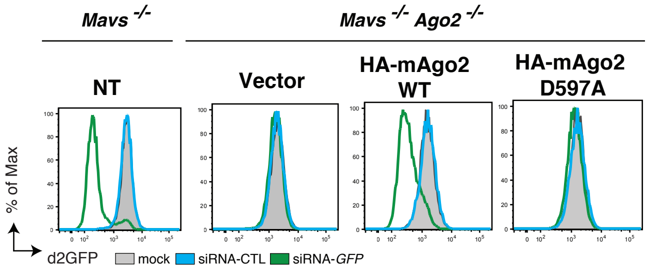

Appendix Figure S5

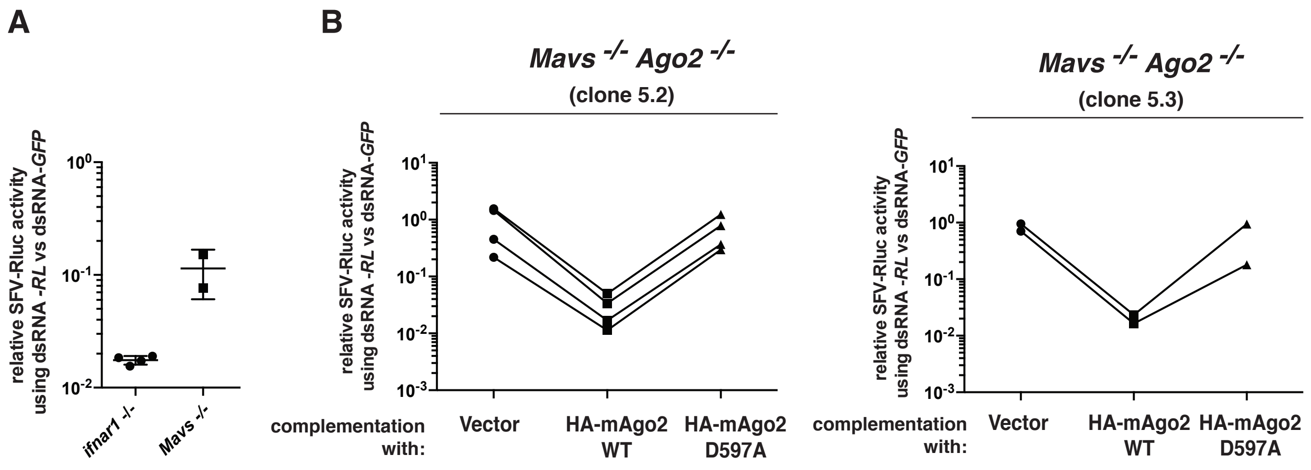

Appendix Figure S6

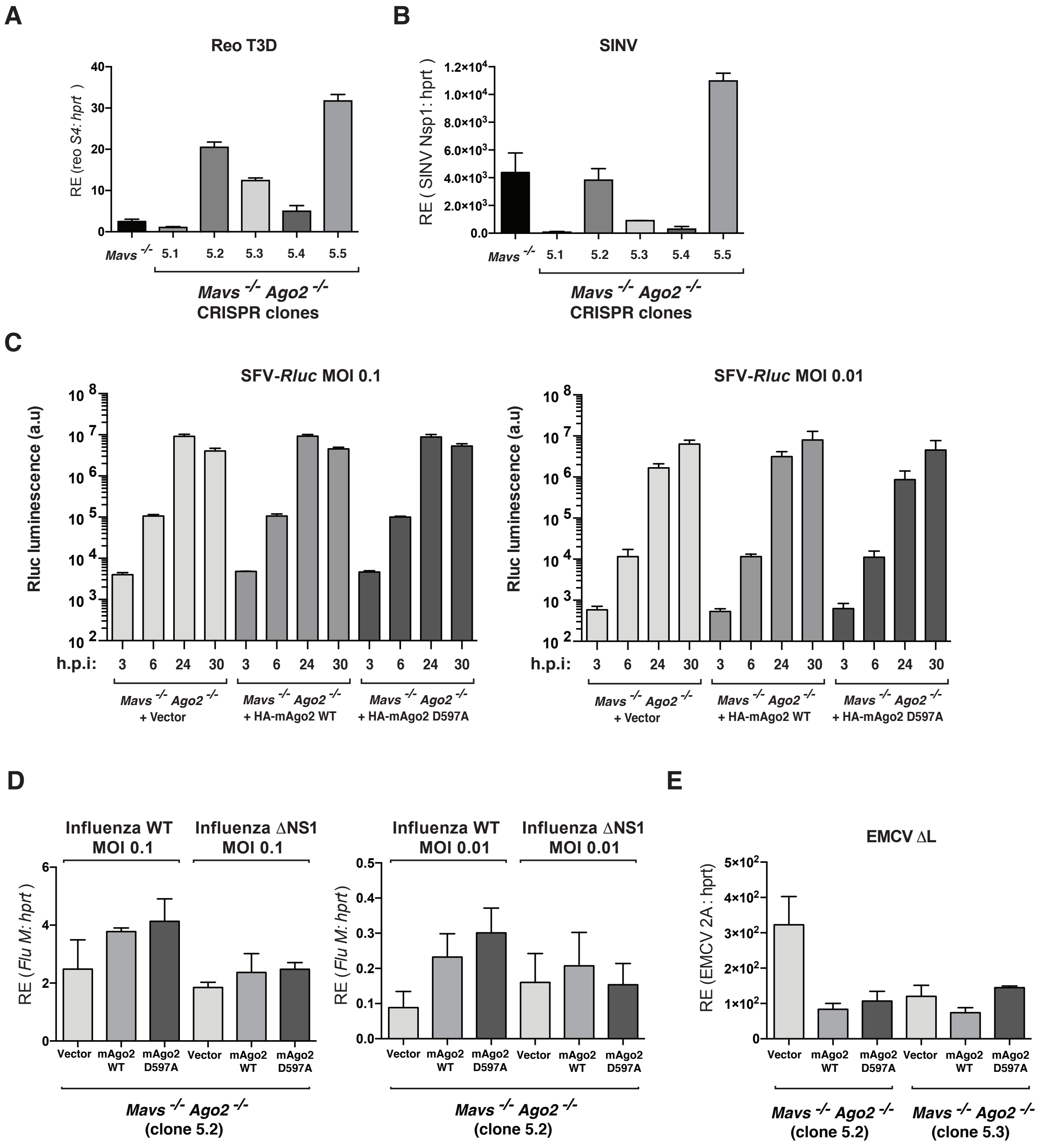

Appendix Figure S7

Appendix Figure legends

Appendix Figure S1. Cytotoxicity induced by transfection of long dsRNA. (A) mESCs or **(B)** L929 cell numbers after dsRNA or mock (–) transfection at various time-points as indicated. Data show mean and SD of 2 independent experiments.

Appendix Figure S2. Sequence-specific gene silencing by long dsRNA in *Mavs*$^{+/-}$ and *Mavs*$^{-/-}$ MEFs. (A) Immunoblot of Mavs and GFP in *Mavs*$^{+/-}$ and *Mavs*$^{-/-}$ MEFs transduced with a GFP-expressing lentiviral vector and FACSsorted for GFP positive cells or left untransduced. β-actin served as loading control. **(B)** *Mavs*$^{+/-}$ and *Mavs*$^{-/-}$ MEFs stably expressing GFP were transfected with the indicated Cy5-labeled dsRNAs or siRNAs. Relative expression (RE) of the interferon-stimulated gene *Ifit1* was assessed 24 hrs post-transfection by qRT-PCR and normalised by *Gapdh*. Graph shows mean and SD of biological duplicates. **(C)** *Mavs* $^{+/-}$ and *Mavs* $^{-/-}$ MEFs cell numbers after dsRNA or mock (–) transfection at various time-points as indicated. **(D)** Same experiment as in (B) but the relative expression of *gfp* was measured and normalised by *Gapdh* 24 hrs (left graph) and 48 hrs (right graph) post-transfection of the indicated dsRNA or siRNA. Data show mean and SD of 2 biological duplicates (B-C) or 2 independent experiments (D).

Appendix Figure S3. Sequence-specific gene silencing induced by siRNAs is not affected by IFN treatment. *Mavs*$^{+/-}$ and *Mavs*$^{-/-}$ MEFs stably expressing d2GFP were stimulated with recombinant IFN A/D (200U/ml) 24 hrs prior transfection with siRNA as indicated. IFN A/D treatment was maintained for 2 days post-transfection and then GFP level was monitored. Histograms plots are representative of 3 independent experiments. Each histogram represents a sample size of 10 000 cells.

Appendix Figure S4. Sequence-specific gene silencing by long dsRNA is Ago2-dependent and Ago1-independent. (A) Immunoblot of Ago2 in parental *Mavs*$^{+/-}$ MEFs stably expressing GFP and in two independent *Mavs*$^{+/-}$ *Ago2*$^{-/-}$ and *Mavs*$^{-/-}$ *Ago2*$^{-/-}$ clones generated by CRISPR/CAS9-mediated genome engineering using two sets of short guide RNAs (sgRNA #1 and sgRNA #2). Some clones (clones 1.3 and 2.1) retained wild type expression of Ago2. P97 served as loading control. **(B)** Two *Mavs* $^{+/-}$ *Ago2* $^{-/-}$ and two *Mavs* $^{-/-}$ *Ago2* $^{-/-}$ clones (from distinct sets of sgRNAs) were transfected with the indicated siRNA (top plots) or dsRNA (bottom plots) and GFP level was measured 3 days later. Clones 1.3 and 2.1, which retained wild type expression of Ago2, as mentioned in (A), were also analysed. **(C)** Same cells described in (B) were transfected with the indicated dsRNA and GFP level was analysed 3 days later. **(D)** Immunoblot of Ago1 in a *Mavs*$^{-/-}$ *AGO1* $^{-/-}$ clone (# 4.2) generated by CRISPR/CAS9-mediated genome engineering versus a clone (# 4.1) that retained wild type expression of Ago1. P97 served as loading control. **(E)** Clones from (D) were transfected with the indicated siRNA and GFP level measured 3 days later. **(F)** Clones from (D) were transfected with the indicated dsRNA and GFP level was analysed 3 days later. All histograms plots are representative of 2 independent experiments. Each histogram represents a sample size of 10 000 cells.

Appendix Figure S5. Ago2 catalytic activity is essential for silencing mediated by siRNA. *Mavs*$^{-/-}$ *Ago2*$^{-/-}$ clones (clone 5.2) stably expressing d2GFP and transduced with the empty vector or a vector expressing HA-tagged wild-type (HA-mAgo2 WT) or catalytic mutant version of mouse Ago2 (HA-mAgo2 D597A) were transfected with the indicated siRNA and d2GFP was analysed 2 days later. Histograms plots are

representative of 2 independent experiments. Each histogram represents a sample size of 10 000 cells.

Appendix Figure S6. Introduction of dsRNA in IFN-deficient cells provides sequence-specific antiviral activity.

(A) Relative SFV-Rluc activity at 24 h.p.i in *Ifnar1*$^{-/-}$ or *Mavs*$^{-/-}$ MEFs previously transfected with dsRNA-*RL* versus dsRNA-*GFP* as described in Fig 6A and Fig 6C. Each dot represents an independent experiment **(B)** Same as in (A) but with *Mavs*$^{-/-}$ *Ago2*$^{-/-}$ clones (clone 5.2 and 5.3) individually complemented to express wild-type (HA-mAgo2 WT) or a catalytic mutant (HA-mAgo2 D597A) version of mAgo2 or transduced with a control vector as described in Fig 6D.

Appendix Figure S7. RNAi does not appreciably impact antiviral resistance in unvaccinated IFN-deficient cells.

(A) Parental Mavs$^{-/-}$ MEFs and 5 different Mavs$^{-/-}$ Ago2$^{-/-}$ clones (clone 5.1-5.5) were infected with Reovirus strain type 3 Dearing (Reo T3D) at an MOI 0.01. The relative level of reovirus gene segment S4 genome was measured at 24 h.p.i. and normalised to *Hprt*. Data show mean and SD of 2 biological duplicates. **(B)** Same cells as in (A) were infected with Sindbis virus (SINV) at an MOI 0.01. The relative level of SINV *Nsp1* gene was measured at 24 h.p.i. and normalised to *Hprt*. Data show mean and SD of biological duplicates. **(C)** *Mavs*$^{-/-}$ *Ago2*$^{-/-}$ clones (clone 5.2) expressing d2GFP and transduced with the empty vector or a vector expressing HA-tagged wild-type (HA-mAgo2 WT) or catalytic mutant version of mouse Ago2 (HA-mAgo2 D597A) were infected with an MOI 0.1 (left panel) or an MOI 0.01 (right panel) of SFV-Rluc. *Renilla* luciferase activity was measured 24 hours later. Each bar represents mean +

SD of biological triplicates. The results are representative of 2 independent experiments. **(D)** Same cells as in (C) were infected with Influenza A/PR/8/34 WT and Influenza A/PR/8/34 ΔNS1 at MOI 0.1 or 0.01 and the relative level of M segment was measured at 24 h.p.i. and normalised to *Hprt*. Data show mean and SD of biological triplicates. **(E)** Same as in (C) but cells were infected with EMCV ΔL at MOI 0.001. Data show mean and SD of biological duplicates.

Appendix Table S1. List of Primers used in this study

Probes for Northern analysis

Description	Primer name	Primer sequence (5' to 3')
U6	U6_NB_probe	GCAGGGGCCATGCTAATCTTCTCTGTATCG
miRNA marker	miRNAmarker_NB_probe	AAATCTCAACCAGCCACTGCT
GFP	GFP_T7_IVT_sense_Fwd	GAAATTAATACGACTCACTATAGGGAGAATGGTGAGCAAGGGCG
	GFP_IVT_sense_Rev	AAGCACTGCACGCCGTAGG
RL	RL_T7_IVT_sense_Fwd	GAAATTAATACGACTCACTATAGGGAGAATGACTTCGAAAGTTTATG
	RL_T7_IVT_sense_Rev	TGGCTCAATATGTGG

Primers used for *in vitro* transcription

Description	Primer name	Primer sequence (5' to 3')
GFP-sense	GFP_T7_IVT_sense_Fwd	GAAATTAATACGACTCACTATAGGGAGAATGGTGAGCAAGGGCG
	GFP_IVT_sense_Rev	AAGCACTGCACGCCGTAGG
GFP-antisense	GFP_T7_IVT_antisense_Fwd	GAAATTAATACGACTCACTATAGGGAGAAAGCACTGCACGCCG
	GFP_T7_IVT_antisense_Rev	ATGGTGAGCAAGGGCGAGGAGC
RL-sense	RL_T7_IVT_sense_Fwd	GAAATTAATACGACTCACTATAGGGAGAATGACTTCGAAAGTTTATG
	RL_IVT_sense_Rev	TGGCTCAATATGTGG
RL-antisense	RL_T7_IVT_antisense_Fwd	GAAATTAATACGACTCACTATAGGGAGATGGCTCAATATGTGG
	RL_T7_IVT_antisense_Rev	ATGACTTCGAAAGTTTATG

Primers used for cloning and site-directed mutagenesis

Description	Primer name	Primer sequence (5' to 3')
To clone d2GFP into pRRLsin_PGK plasmid	d2GFP_ cloning_Fwd	CGCGGATCCACCGGTCGCCACCATGGTGAGCAAGGGC
	d2GFP_ cloning_Rev	ACGCGTCGACGCGGCCGCTTTACACATTGATCCTAGC
To clone HA-mAgo2 into pLHCX plasmid	HA-mAGO2 cloning_Fwd	CCCAAGCTTGCCACCATGTACCCTTATGACGTGCCCGATTACGCTATGTACTCGGGAGCC
	mAGO2 cloning_Rev	CCATCGATTCAAGCAAAGTACATGG
To insert mutation D597A into mAgo2	mAGO2 D597A_Fwd	CATCTTCCTGGGAGCCGCTGTCACCCACCCACC
	mAGO2 D597A_Rev	GGTGGGTGGGTGACAGCGGCTCCCAGGAAGATG

Primers used for qRT-PCR

Description	Primer name	Primer sequence (5' to 3')
S4 segment reovirus T3D	Reo_S4 qRT_Fwd	CGCTTTTGAAGGTCGTGTATCA
	Reo_S4 qRT_Rev	CTGGCTGTGCTGAGATTGTTTT
NSP1 gene of Sindbis virus	Sindbis_NSP1 qRT_Fwd	CACTCCAAATGACCATGC
	Sindbis NSP1 qRT_Rev	GGTGCTCGGAAAACATTC
M gene of Influenza virus PR8	INF_NSP1 qRT_Fwd	CAAGCAGCAGAGGCCATGGA
	INF_NSP1 qRT_Rev	GACCAGCACTGGAGCTAGGA
2A gene of EMCV	EMCV_2A qRT_Fwd	AGGCGGTTCTAAGAGCAGAACCAT
	EMCV_2A qRT_Rev	AGTGGGCATTGAAGATCCGGTACA
HPRT	HPRT_ qRT_Fwd	TGAAGAGCTACTGTAATGATCAGTCAAC
	HPRT_ qRT_Rev	AGCAAGCTTGCAACCTTAACCA

Primers used for CRISPR/Cas9-mediated genome engineering

Description	Primer name	Primer sequence (5' to 3')
CRISPR for mouse Ago2, target in exon 2	mAgo2_exon2_CRISPR_Fwd	CACCGTGGTGCCGAAGTCCGGCCG
	mAgo2_exon2_CRISPR_Rev	AAACCGGCCGGACTTCGGCACCAC
CRISPR for mouse Ago2, target in exon 4	mAgo2_exon4_CRISPR_Fwd	CACCGATCGTCTCGAAGGGGACGC
	mAgo2_exon4_CRISPR_Rev	AAACGCGTCCCCTTCGAGACGATC
CRISPR for mouse Ago1, target in exon 2	mAgo1_exon2_CRISPR_Fwd	CACCGATTGACGTTTACCATTACG
	mAgo1_exon2_CRISPR_Rev	AAACCGTAATGGTAAACGTCAATC
