# Supplementary figures and images for: Inactivation of the type I interferon pathway reveals long double‐stranded RNA‐mediated RNA interference in mammalian cells
Source: EMBO J. 2016 Nov 4;35(23):2505–18. doi: 10.15252/embj.201695086 (PMC5167344; doi:10.15252/embj.201695086)

**A**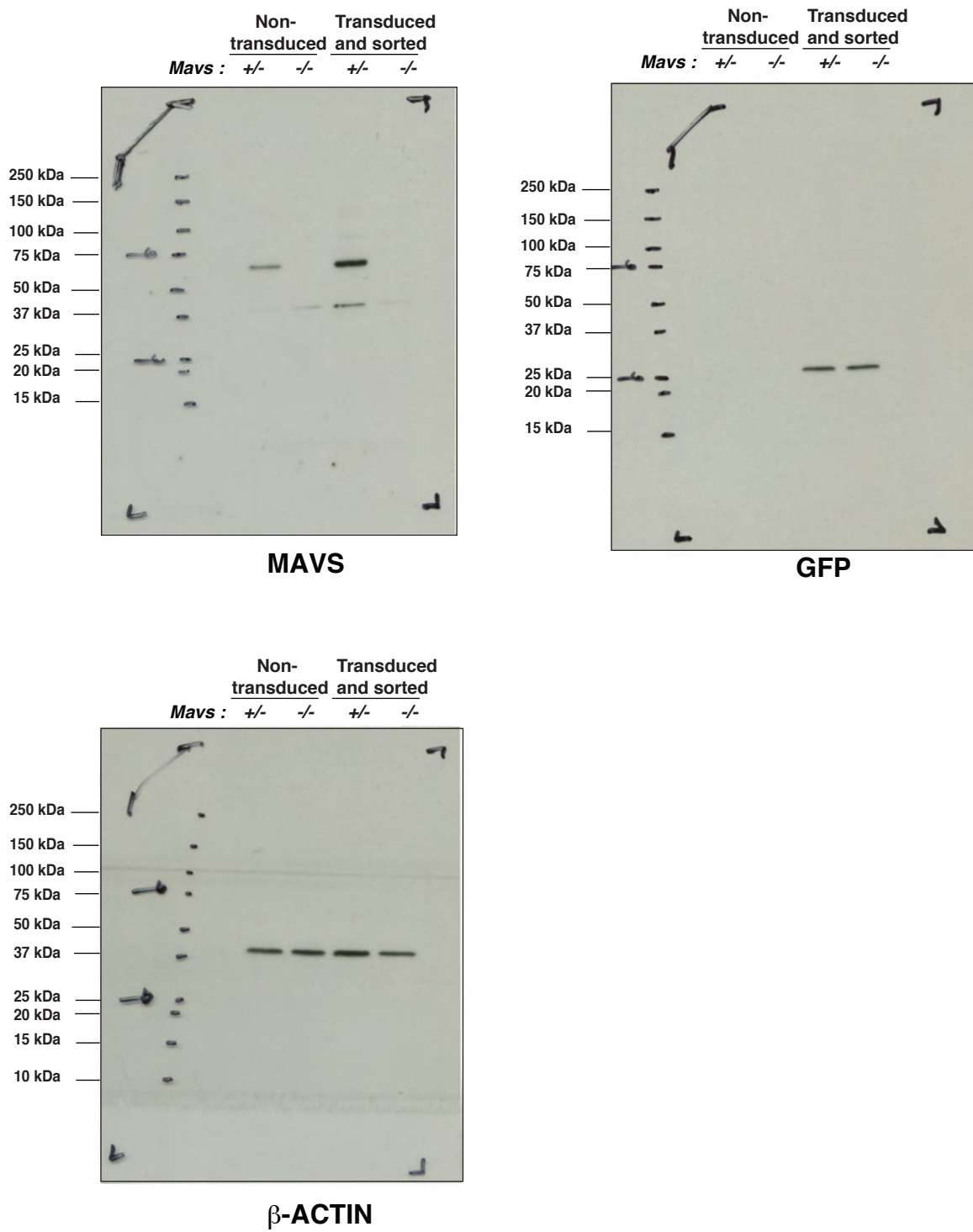**Appendix Figure S2- Source data**

Supplement: Supplementary file 2 — Source Data for Appendix [file EMBJ-35-2505-s002.zip › EMBOJ_95086_Source_Data_for_Appendix/Source_Data_for_Appendix_Figure_S2.pdf]

**A**

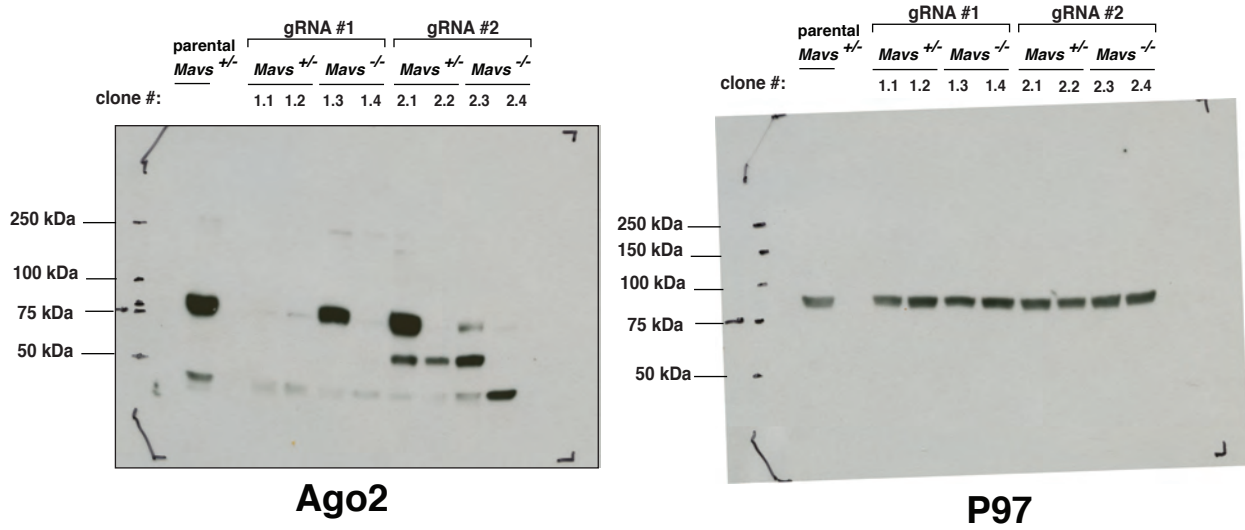

**D**

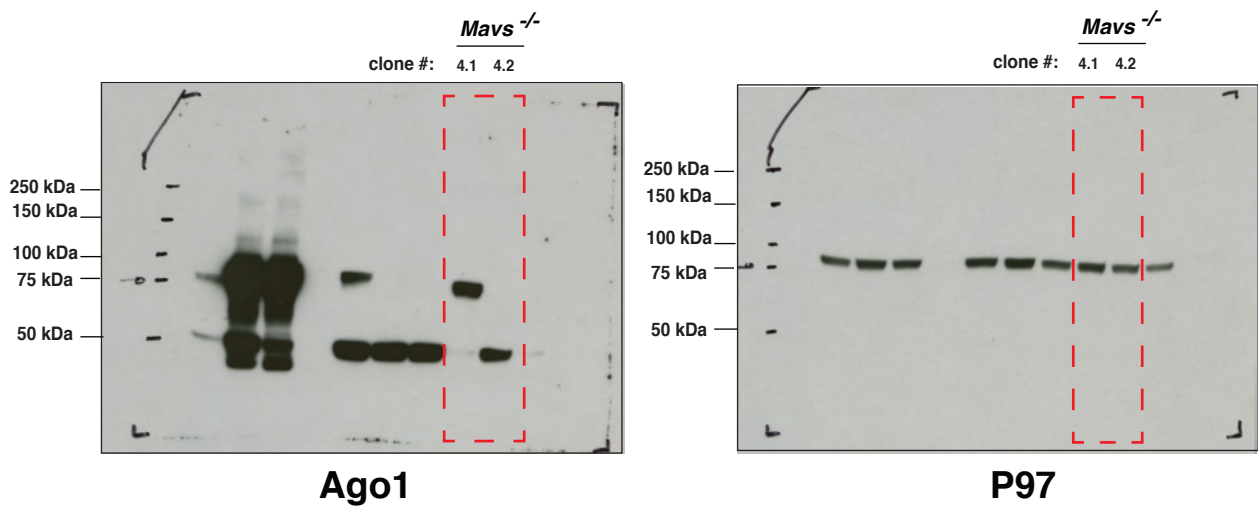

**Appendix Figure S4- Source data**

Supplement: Supplementary file 2 — Source Data for Appendix [file EMBJ-35-2505-s002.zip › EMBOJ_95086_Source_Data_for_Appendix/Source_Data_for_Appendix_Figure_S4.pdf]

**A**

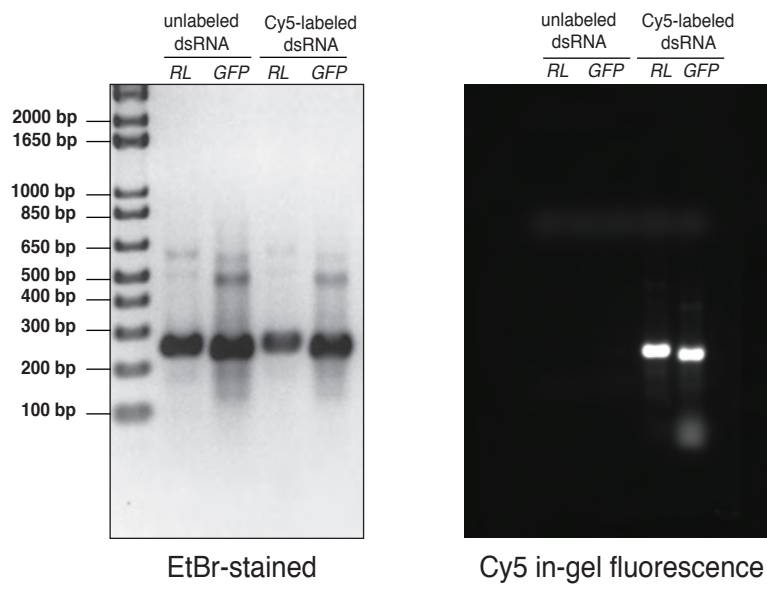

**Figure 1- Source data**

Supplement: Supplementary file 4 — Source Data for Figure 1 [file EMBJ-35-2505-s003.pdf]

**A**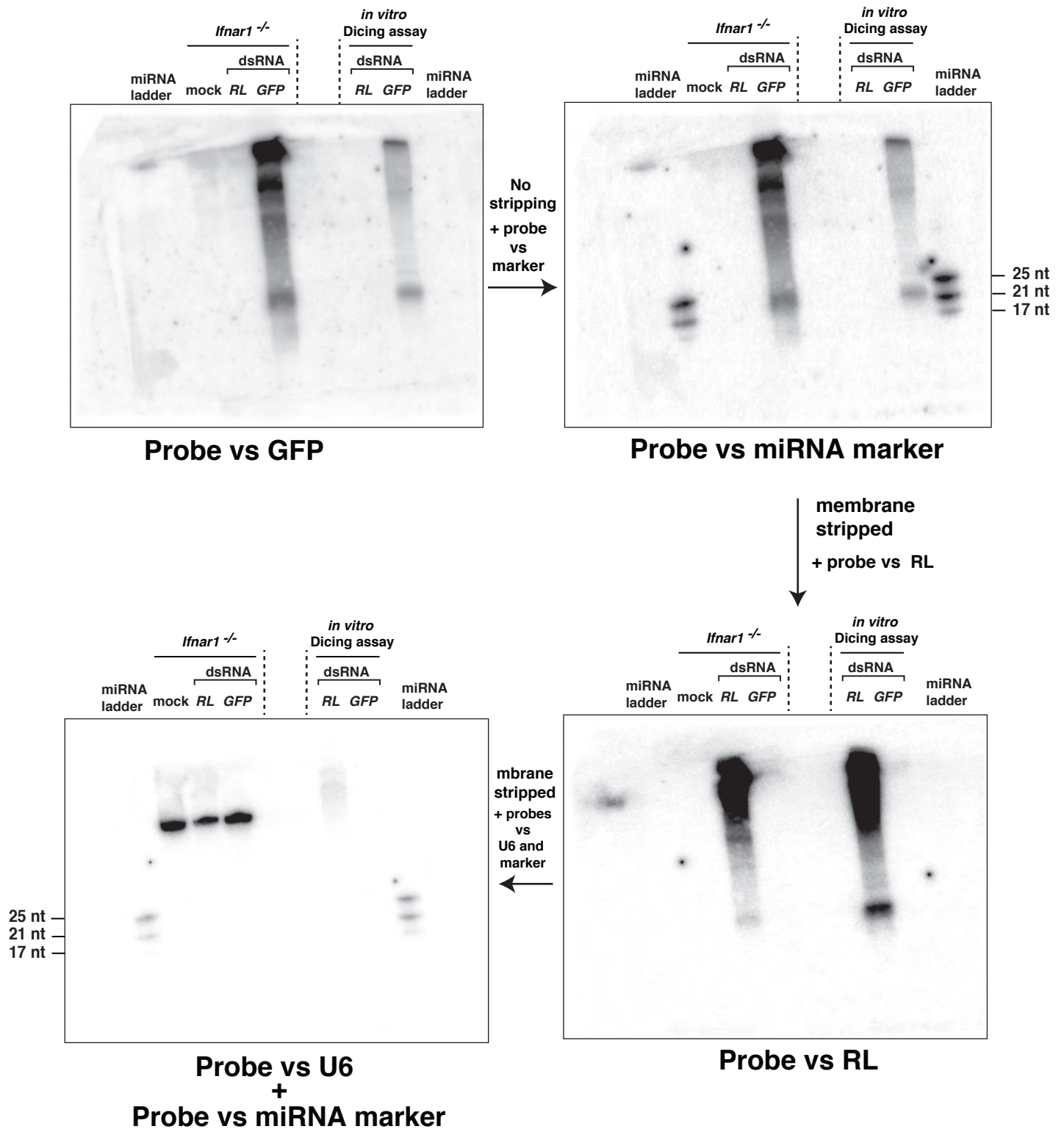**Figure 4- Source data**

**B**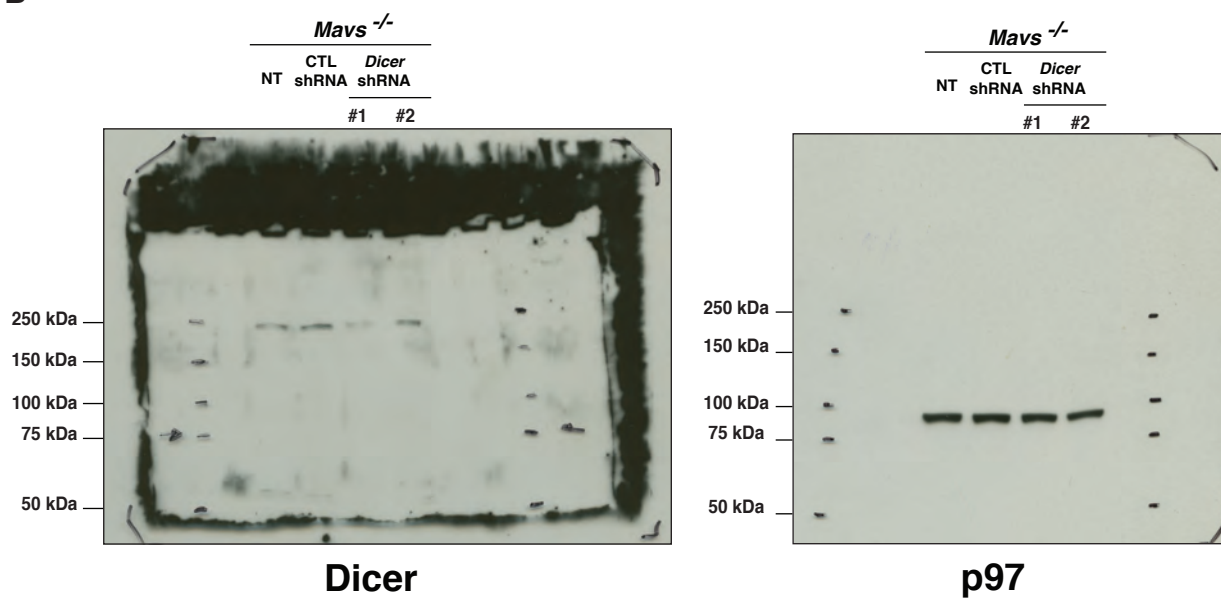**D**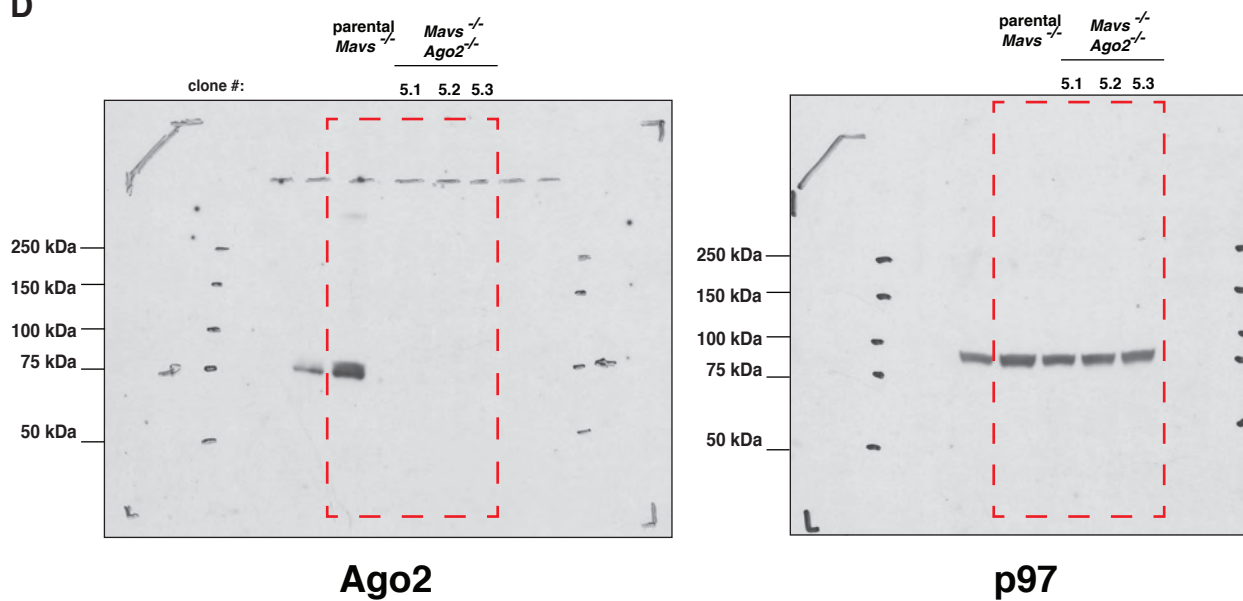**Figure 4- Source data**

Supplement: Supplementary file 5 — Source Data for Figure 4 [file EMBJ-35-2505-s004.pdf]

**A**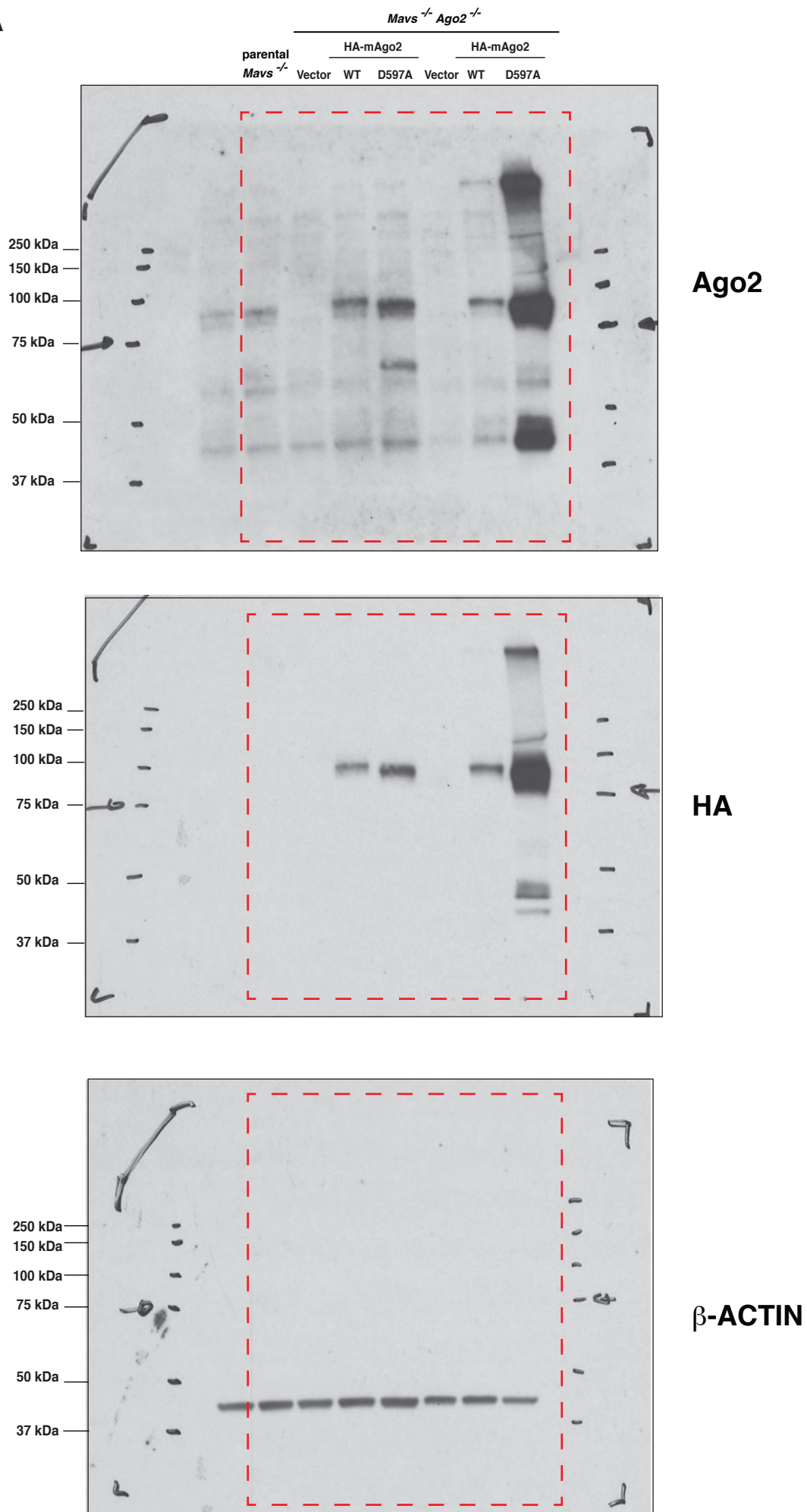**Figure 5- Source data**

Supplement: Supplementary file 6 — Source Data for Figure 5 [file EMBJ-35-2505-s005.pdf]
